# Optimizing the acceleration of Cheddar cheese ripening using response surface methodology by microbial protease without altering its quality features

**DOI:** 10.1186/s13568-021-01205-9

**Published:** 2021-03-22

**Authors:** Amaal Mohammed Alhelli, Nameer Khairulla Mohammed, Eilaf Suliman Khalil, Anis Shobirin Meor Hussin

**Affiliations:** 1 Department of Water Resource Technique, Institute of Technology, Middle Technical University, Alzafaranya, Baghdad 29008 Iraq; 2 Food Science and Biotechnology Department, Faculty of Agriculture, Tikrit Uiversity, Tikrit, 34001 Iraq; 3 Department of Dairy Production, Faculty of Animal Production, University of Khartoum, Khartoum, Sudan; 4 Faculty of Food Science and Technology, Universiti Putra Malaysia, 43400 Serdang, Selangor Darul Ehsan Malaysia; 5 Halal Products Research Institute, University Putra Malaysia, 43400 Serdang, Selangor Darul Ehsan Malaysia

**Keywords:** *Penicillium candidum*, Ripening time, Protease, Cheddar cheese, RSM

## Abstract

Cheddar cheese proteolysis were accelerated employing *Penicillium candidum* PCA1/TT031 protease into cheese curd. In the present study, several of the significant factors such as protease purification factor (PF), protease concentration and ripening time were optimized via the response surface methodology (RSM). The ideal accelerated Cheddar cheese environment consisted of 3.12 PF, 0.01% (v/v) protease concentration and 0.6/3 months ripening time at 10 °C. The RSM models was verified to be the most proper methodology for the maintain of chosen Cheddar cheese. Under this experimental environment, the pH, acid degree value (ADV), moisture, water activity (a_w_), soluble nitrogen (SN)%, fat and overall acceptability were found to be 5.4, 6.6, 35%, 0.9348, 18.8%, 34% and 13.6, respectively of ideal Cheddar cheese. Furthermore, the predicted and experimental results were in significant agreement, which confirmed the validity and reliability of the suggested method. In spite of the difference between the ideal and commercial Cheddar cheese in the concentration of some of amino acids and free fatty acids, the sensory evaluation did not show any significant difference in aroma profile between them.

**Supplementary Information:**

The online version contains supplementary material available at 10.1186/s13568-021-01205-9.

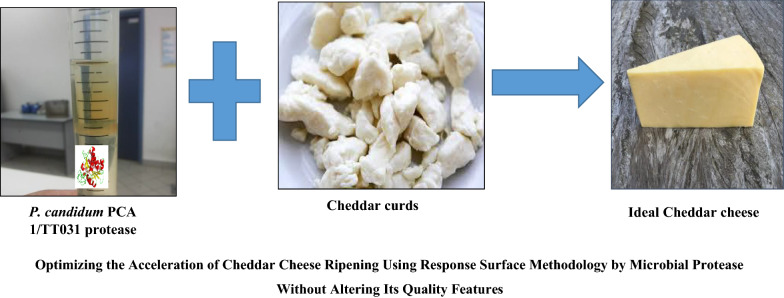

## Key points

- *P. candidum* PCA1/TT031 protease was approved hasten the maturation of Cheddar cheese.
- These results approved the “component balance theory”, which is attributed the flavour of cheese to an extensive range of sapid and aromatic compounds.
- RSM approved gain the ideal flavour of Cheddar cheese via combining practical and theoretical process.

## Introduction

Cheese is one of the fermented milk-based foods indicated by its various texture, aroma and flavor. Maturing is the greatest essential industrial stage in cheese production, establishing a cascade of biochemical results, resulted by a varied array of microbial flora that allow the observed sensory characteristics. These sensory attributes are assessed by diverse instrumental, descriptive and computational approaches (Khattab et al. 2019).

In terms of overall consumption and volume, Cheddar is the largest variety of cheese in the world (Batool et al. 2018). Cheddar is widespread all over the world due to its distinctive flavor characteristics that are produced during the ripening. Ripening is a process in which numerous biochemical transformations take place that switch chalky curd to a flavorful cheese (Khan et al. 2019). Metabolic and Biochemical processes lead to the considerable changes in texture and flavor of cheese. These developments are generally indicated as lipolysis, glycolysis and proteolysis. Lactose is metabolized into citrate diacetyl, lactic acid and alcohol. Cheddar cheese ripening is a time taking process, period of the ripening may be as long as 1 year (Nateghi 2017).

Long maturation time is a big interruption in the acceptance of cheese in developing countries. Additional, energy supplies in these countries are either very expensive or insufficient (Luo et al. 2019). Consequently, those techniques of cheese ripening should be detected which can considerably decrease the ripening time without reduce the quality attributes of Cheddar cheese. The researchers worldwide have explored for solutions to accelerate the ripening process of semi-hard and hard cheese applying different means and procedures in order to decrease the production value and hasten the capital sequence, all presenting differing levels of achievement, such as, utilizing enzymes and elevated temperatures (Karaca and Güven 2018; Ha 2019).

Batool et al. (2018) maturated the Cheddar cheese at elevated temperature via addition of vitamin E and selenium, they found that the Cheddar cheese ripening may be decrease to 6 weeks by elevated temperature (18 °C) using vitamin E and selenium. Walsh et al. (2020), demonstrated that aged white Cheddar storage at a higher temperature was distinguish similarly by consumers as one stored for 1 year at a slightly lower temperature. Hannon et al. (2006) discovered adding of 0.25 or 1 g/100 g of enzyme-modified cheese (EMC) particles to Cheddar cheese curd when manufactured had a favourable influence on the enhancement of cheese flavour up to 4 month of maturing in comparison with the control (which is 6 months).

To optimize the conditions of acceleration Cheddar cheese, to decrease their costs, and the amount of time and work, several theoretical methods have been used. Such as, the Response Surface Methodology (RSM) is recognized as the collecting of mathematical and statistical analysis that is effective for assessment and modelling in applications where a response of dependent (or interest) is impacted by different factors (Singh et al. 2013; Zhao et al. 2020). Then again, there is limited information about hastening Cheddar cheese ripening using purified extracellular enzymes. Therefore, the scope of this current research was to study the effect of protease produced by *P. candidum* PCA1/TT031 on the chemical and physical properties of Cheddar cheese prepared from cow milk and the influence of this enzymes on increased rate of improvement on Cheddar cheese maturation process using RSM. The assessment was performed in association with commercially produced Cheddar cheese.

## Materials and methods

### Preparation of *P. candidum* PCA1/TT031 protease

The pure protease of *P. candidum* PCA1/TT031 [Commercially freeze–dried strains obtained from Chr. Hansen which is the commercial names of SWING® PCA1/10U; (Arpajon, France)] were prepared according to Alhelli et al. (2016) by an Aqueous Two-Phase System. The best protease purification could be achieved under the conditions of 9.0% (w/w) PEG 8000, 15.9% sodium citrate concentration, and 5.2% NaCl, which achieved in protease partitioning of a one-sided for the bottom phase with a 6.8-fold protease purification factor, a partition coefficient of 0.2, and a yield of 93%.

### Production of Cheddar cheese

#### Design of experiment

In order to determination of the optimized maturation process for accelerating Cheddar cheese ripening via purified microbial protease from the *P. candidum* PCA1/TT031 solution, we employed the design methodology for obtaining the experimental space enclosing the preselected conditions. After that we investigated the physicochemical, aroma and sensory profile. We examined the effects of three independent variables, i.e., purification factor of protease (3.12–6.46, X1), protease concentration [0.01–0.04% (w/w), X2] and maturation time (0–3, X3) and their influence was detected on the pH (Y1), fat (Y2), moisture (Y3), acid degree value (Y4), soluble nitrogen (Y5), fat (Y6) and overall acceptability (Y7) for the ripened cheeses by the, *P. candidum* PCA 1/TT031 protease. These factors were optimized using the RSM model. In this research, we have employed the Central Composite Design (CCD) for assessing and approximating the total quadratic design model for each response. Twenty trials for all the three variables were measured with every variable being assessed at five different levels (Additional file 2: Table S1). The information achieved was calculated graphical manner and in a statistically by utilizing the statistical software of Minitab v16 (Minitab Inc., State College, PA, USA). The software assisted in creating a mathematical design model for establishing the likely response for the data obtained.

#### Cheese making

Commercial rennet was diluted 1 part enzyme in 50 parts de-ionized water prior to use based on the product information sheet. Cheddar cheese was made using the process described by (Sood and Kosikowski 1979). Twenty sets of Cheddar cheese were prepared from cow’s fresh milk. Measured amounts of *P. candidum* PCA 1/TTO31 at a concentration of 0.01–0.04% (v/v) and purification factor (Pf) 3.12–6.46 (prepared according to preliminary study) were mixed with the salt and added to the milled curd (Additional file 2: Table S1). At last, cheeses were permitted to get dried at room temperature for 10 min prior to vacuum packing; further, the pressed cheeses were vacuum packed in nylon bags which are vacuumed by employing a vacuum packaging machine (model vac master, Kansas, USA). All cheese testing and analysis were conducted at day 0, 1, 2, and 3 months of maturation at 10 °C. Free amino acids (FAA), free fatty acids (FFA) and aroma attributes of ideal treatment were contrast with a positive control (commercial Cheddar cheese).

#### Variance analysis

To find out the significant variables in addition to LSD or to measure the least significance tests to assess the difference amongst the examined samples, the Analysis of Variance (ANOVA) technique was employ. Each sample was subjected to twofold or threefold value and the following data was recorded and documented as the mean ± SD of independent tests. analysed the consequence of the suggested model was analysed by Fisher’s test (F-test). Moreover, the regression equations were summated to the F-test, to calculate the coefficient of determination (*R*^2^). The corresponding polynomial equation was expressed as three-dimensional surface plots to predict the relationships between the dependent variables (responses) and the experimental levels of the independent variables (different factors) involved in the design.

#### Optimization and validation of the experimental process

To approve suitability and validity of the model, various tools (graphical and numerical) of the optimised parameters were examined experimentally (Xu et al. 2013). Moreover, predicted values gained from the regression equation were contrasted with the experimental values and the response surface model sufficiency was verified (Mirhosseini et al. 2008; Xu et al. 2013).

#### Compositional analysis

Fat and moisture content in cheese were assessed at 0, 1, 2 and 3 months by the standard methods (Horwitz 2010). The pH of cheese samples was measured using a digital pH meter (Mettler—Toledo, GmbH; 8603 Schwarzenbach, Switzerland) (Ruyssen et al. 2013). Water activity (a_w_) of cheese samples were measured using aqua lab water activity analyzer (Washington, USA). The value of acid degree (ADV) is established using the process Bureau of Dairy Industry (Deeth 1976) with slight modification. The soluble nitrogen (SN) content was determined based on the method described by (Kuchroo and Fox 1982). SN (%) quantified as percentage of total nitrogen (SN, % of TN). The cheese samples were analysed in triplicate.

#### Free amino acid composition

The free amino acid constitution of commercial Cheddar cheese and the best cheese variety which resulted using PF 5.85, Conc. of pure protease 0.01% (v/v) and 0.6 month ripened at 10 °C were assessed by using a system called gradient HPLC (Jasco CO-2065 Plus) with pre-column phenylisothiocyanate (PITC) derivatisation (Rozan et al. 2000). Concentration of every amino acid was denoted as mg/g of sample.

### Volatile flavor compounds release in cheese

#### Sample preparation (SPME procedures)

The static headspace solid-phase micro-extraction method (HS-SPME) were used to remove the volatile elements (Verzera et al. 2010). The ideal cheese using PF 3.12, Conc. of pure protease 0.01% (v/v) and 0.6/3 months ripened at 10 °C and commercial Cheddar cheese were analysed for fragrance volatiles by the SPME–GC–MS technique.

#### GC–MS analysis

The preliminary identification and verification of the volatile components were conducted with a Hewlett-Packard 6890N GC device (Agilent Technologies, Wilmington, DE) furnished with Time-of-Flight Mass Spectrometer (TOFMS), (Pegasus III, LECO Corp., St. Joseph, MI, USA). After verification analysis, the volatile components were then measured using a Hewlett-Packard 6890 GC system stocked with an HP-Innowax (60 m, 0.25 mm, 0.25 μm) capillary column (J and W Scientific, Folsom, CA, USA). The fatty acid solutions (FA) like ethanol, acetic acid, 1,3-butanediol, 2,3-butandiol, [*S*-(R*, R*)], hexadecanoic acid, ammonium acetate, hexanoic acid-methyl ester, pentanoic acid and butanoic acid were computed as relative total peak areas percentage of all the fatty acids in the sample of cheese on the basis of the FA peak areas that were present in the chromatogram.

#### Aroma profile

A single, high quartz, uncoated surface acoustic wave (SAW) resonator electronic nose was employed in order to estimate the profile of aroma of the new ideal Cheddar cheese and industrial Cheddar cheese. The aroma present in the cheeses was assessed according to the procedure described by Manaf et al. (2008) with an ultrafast GC known as zNose 7100 analyser (Electronic Sensor Technology Co., Newbury Park, CA, USA). The aroma component profiles taken from analysis were showed as zNose chromatogram and Vaporprints™ (polar plots).

#### Sensory evaluation

Overall acceptability (flavour, aroma and texture attributes) of the processed, ideal and commercial cheeses during the maturation period was conducted according to Jung et al. (2013a) and with few modifications in the processes. The research consisted of 15 trained Iranian and Arabian panellists accustomed to Cheddar cheese. Overall acceptability was evaluated on the scale of 1 to 10 with 10 denoting ‘extremely like’ and 1 representing ‘extremely dislike’. Before every session, the Cheddar cheese purchased from the manufacturing firm was presented to get habituated with the sensory characteristics of the cheese (Jung et al. 2013a). The values of each sample were averaged over all panellists. The sensory assessment was conducted in duplicate.

#### Statistical data analysis

Samples were gathered randomly and information was tabulated and put through analysis of variance (ANOVA) and MANOVA (PROC GLM) with MINITAB statistical software, release 16 (MINITAB Inc., state college, PA and USA). In the chromatography assessment, the highest area of each component was regarded as the response variables, whereas the storage time, purification factor and concentration of enzyme were considered as the separate variables in this research. The Two-Sample test assessment was used to establish the non-significant or significant interaction effect of separate variables on response variable on FFA, FAA and aroma components of ideal and industrial Cheddar cheese. The resultant variables have more significance (P ≤ 0.05) in case the absolute F-ratio becomes greater (Mirhosseini et al. 2008). All measurements were performed in triplicate and denoted as the mean ± SD of independent trials.

## Results

### Optimizing the Cheddar cheese using a response surface methodology

#### The RSM models fitting

The predicted values of the regression coefficients for the RSM models and their corresponding *R*^2^ values were mention (Table 1). As it can be seen from the outcomes, the response variable showed significant (P ≤ 0.05) values for the response surface models and a high *R*^2^ value that was also observed which was in the range of 0.92–0.99. Additionally, Table 2 described the interactive effects and the quadratic model of the Purification factor of protease (X1), protease concentration (X2), ripening time (X3), for every response variable. Also Table 2 shows the significance of the P-value and the F-ratio. It can also be observing that the main influence of PF of protease as the most significant factor (P ≤ 0.05) impact on the most of the responses.

**Table 1:** Regression coefficients, *R*^2^, and probability value of the response surface models

Regression coefficient	pH (Y1)	ADV (Y2)	Moisture (Y3)	a_w_ (Y4)	SN [(%), Y5]	Fat [(%), Y6]	Overall acceptability (Y7)
b0	3.22	22.156	31.27	0.87167	60.4	41.98	32.3
b1	0.96	− 7.752	3.47	0.04122	− 16.8	− 1.75	− 3.0
b2	102.14	− 438.700	− 277.16	− 1.89539	− 1812.0	− 363.49	− 853.9
b3	− 0.71	7.457	− 0.83	− 0.01209	14.3	− 2.40	− 10.0
b1^2^	− 0.10	0.652	− 0.43	− 0.00551	2.0	− 0.02	0.1
b2^2^	− 1629.07	− 764.094	2827.35	− 2.94037	30,030.3	1381.18	14,895.8
b3^2^	− 0.12	− 0.363	0.19	− 0.00385	0.2	− 0.78	0.5
b12	− 11.58	115.769	32.60	0.34731	60.7	53.63	− 9.3
b13	0.00	− 0.474	− 0.08	0.00204	− 2.5	0.71	1.3
b23	22.96	− 52.445	− 67.11	0.23852	26.1	42.82	78.5
*R*^2^	0.99	0.95	0.96	0.94	0.94	0.94	0.92
Regression (*P-*value)	0.00^a^	0.00^a^	0.00^a^	0.00^a^	0.00^a^	0.00^a^	0.00^a^

b0, b1, b2 and b3: the estimated regression coefficient for the main linear effects. b1^2^, b2^2^ and b3^2^: the estimated regression coefficient for quadratic effects. b12, b13 and b23: the estimated regression coefficient for the interaction effects. 1: purification factor of protease (PF), 2: protease concentration (%); 3: ripening time (month)

^a^Significant (P ≤ 0.05)

**Table 2:** The significance of each independent variable effect indicated by using F-ratio and P-value in the final models

Variables	Independent variable	Main effects			Quadratic effects			Interaction effects		
		X1	X2	X3	X1^2^	X2^2^	X3^2^	X1X2	X1X3	X2X3
pH (Y1)	*p*-value	0.002^a^	0.001^a^	0.004^a^	0.001^a^	0.000^a^	0.001^a^	0.004^a^	0.964	0.000^a^
	F-ratio	21.14	28.60	16.55	24.89	43.73	24.00	16.58	0.00	52.63
ADV (Y2)	*p*-value	0.011^a^	0.078	0.006 ^a^	0.020^a^	0.792	0.230	0.007^a^	0.180	0.183
	F-ratio	10.71	4.09	14.02	8.35	0.07	1.69	12.86	2.15	2.13
Moisture (Y3)	*p*-value	0.075	0.112	0.575	0.028^a^	0.195	0.361	0.195	0.751	0.031^a^
	F-ratio	4.19	3.20	0.34	7.16	2.00	0.94	2.00	0.11	6.82
a_w_ (Y4)	*p*-value	0.001^a^	0.044^a^	0.136	0.000^a^	0.781	0.006^a^	0.019^a^	0.123	0.107
	F-ratio	22.59	5.70	2.75	44.44	0.08	14.13	8.63	2.97	3.29
SN [(%), Y5]	*p*-value	0.007	0.003^a^	0.007^a^	0.002^a^	0.001^a^	0.756	0.372	0.005^a^	0.724
	F-ratio	12.74	17.69	13.14	20.47	29.20	0.10	0.90	14.63	0.13
Fat [(%), Y6]	*p*-value	0.138	0.006^a^	0.028^a^	0.866	0.304	0.000^a^	0.006^a^	0.001^a^	0.029^a^
	F-ratio	2.71	13.91	7.17	0.03	1.21	38.69	13.66	23.72	7.03
Overall acceptability (Y7)	*p*-value	0.216	0.003^a^	0.001^a^	0.680	0.000^a^	0.090	0.766	0.003^a^	0.048^a^
	F-ratio	1.80	17.72	28.80	0.18	32.41	3.50	0.10	18.27	5.46

X1, X2 and X3: the main effect of protease purification factor, protease concentration and ripening time X1^2^, X2^2^ and X3^2^: the quadratic effect of protease purification factor, protease concentration and ripening time, respectively. X1X2: the interaction effect of protease purification factor and protease concentration, X1X3: the interaction effect of protease purification factor and ripening time, X2X3: the interaction effect of protease concentration and ripening time

^a^Significant (P ≤ 0.05)

#### pH (Y1)

According on the findings presented in Table 2, it can be observe that the response of the pH (Y1) was significantly (P ≤ 0.05) influenced because of the basic effects of PF of protease (X1), Protease concentration (X2), maturation time (X3) and the quadratic effect of the protease purification factor (X1^2^), protease concentration (X2^2^) and ripening time (X3^2^), with the interaction of the protease purification factor and its concentration (X1X2), the protease concentration, and the ripening time of Cheddar cheese (X2X3), in the studied Cheddar cheeses acceleration. Moreover, it was also seen that the interaction impact of the protease concentration and ripening time significantly affected the pH of Cheddar cheeses (Y1) value. It was also noticed that the quadratic effect of the protease concentration was impact on the pH of Cheddar cheese.

**Fig. 1:**
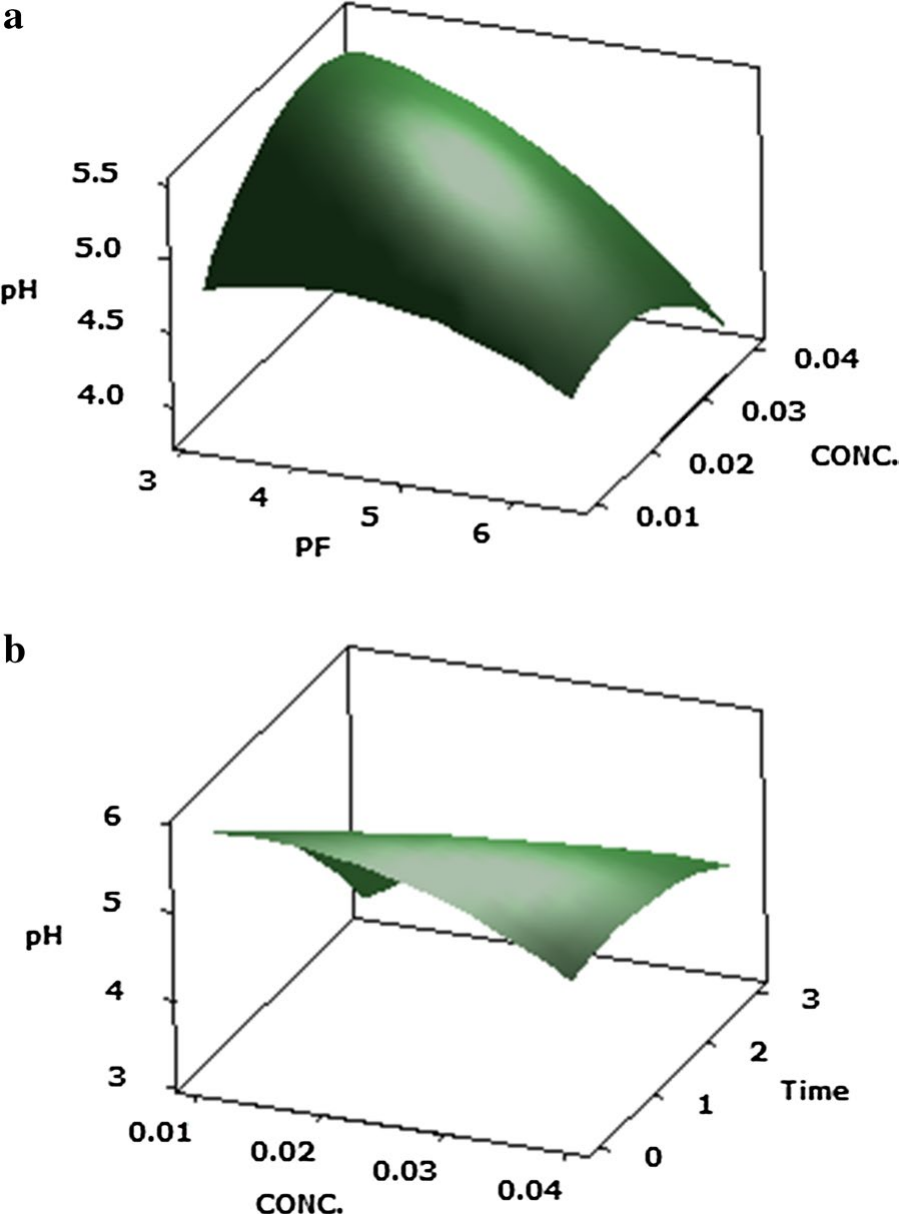
Response surface plots of the central composite design for the interaction effects of **a** PF of protease and protease concentration, **b** PF of protease and ripening time on pH of Cheddar cheese acceleration ripening

From the outcomes obtainable in Fig. 1a, b, it can be seen that the interactions between the purification factor of protease and protease concentration has gain to an increase in the pH value as well as an increase in the protease purification factor and the concentration of protease.

The optimal acceleration Cheddar cheese ripening within the pH (Y1 = 5.4) could be obtained from the following parameters: protease PF 3.12, protease concentration of 0.01% (v/v), and a ripening time of 0.6/3 months.

#### Acid degree value (ADV) (Y2)

**Fig. 2:**
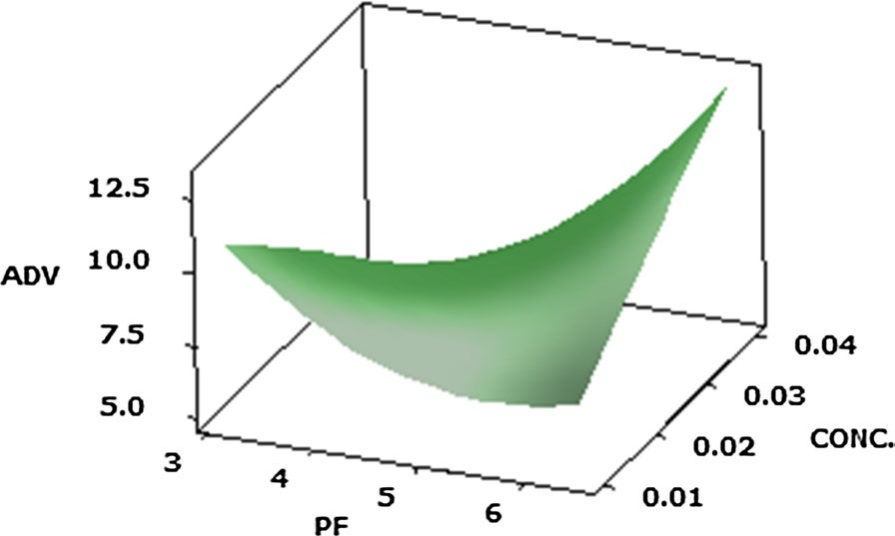
Response surface plots of the central composite design for the interaction effects of PF of protease and concentration of protease on purification factor of protease on ADV of Cheddar cheese acceleration ripening

The use of exo-enzyme *P. candidum* PCA1/TT031 protease to hasten Cheddar cheese maturation produced in ADV values. Alterations in ADV during cheese storage are displayed in Table 2. ADV was prominent (P-value) in all batches of cheese during ripening by the primary effect of independent factors and their interaction. ADV were significantly (P ≤ 0.05) influenced by the main effects of the purification factor of protease and the time of ripening besides the quadratic effect of the purification factor of protease and the interactions between the purification factor of protease and ripening time. Moreover, it was also seen that the interaction effect of the protease PF and protease concentration significantly influenced the ADV of Cheddar cheeses (Y2) value (Fig. 2). In addition, it was noticed that the main effect of the ripening time was influence on the ADV of Cheddar cheese (Y2 = 6.6).

#### Moisture (Y3)

In this paper, it was noticed that moisture was significantly (P ≤ 0.05) affected by the preconditions of the ripening Cheddar cheese with regard to the protease PF (X1), its concentration (X2) and ripening time (X3), along with the quadratic effects of the protease PF (X1^2^) and the interactive effects of the protease concentration and ripening time (X2X3). The interactive effects of the concentration of protease and ripening time exhibited the greatest effect (P ≤ 0.05) on moisture content of Cheddar cheese (Y3) (Table 2).

**Fig. 3:**
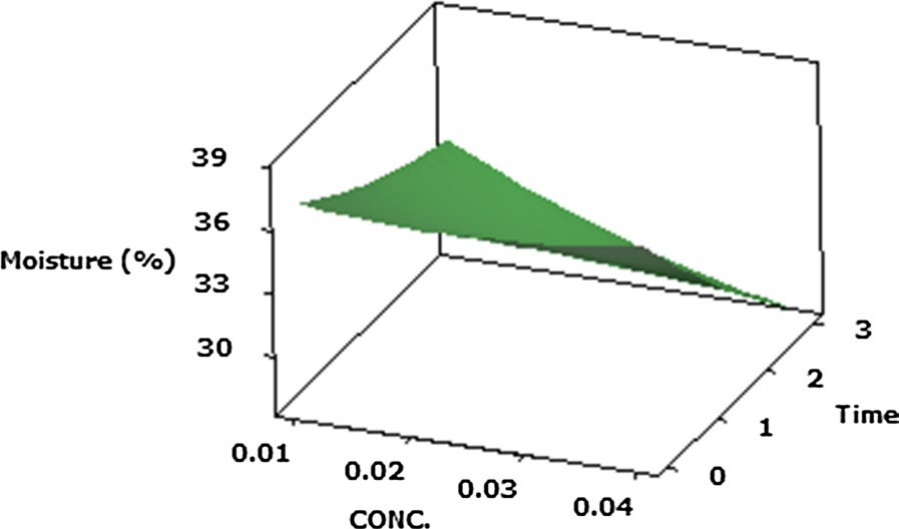
Response surface plots of the central composite design for the interaction effects of protease PF and ripening time on moisture of Cheddar cheese acceleration ripening

The Fig. 3 display the interactive effects of the response variables on the moisture content of Cheddar cheese, whereas the plot shows the maximal moisture in the Cheddar cheese. The optimum accelerated Cheddar cheese moisture (Y3 = 35%) using the *P. candidum* PCA1/TT031 protease was achieved under the following conditions—PF of protease of 3.12; protease concentration of 0.01%; and ripening time of 0.6/3 months.

#### Water activity a_w_ (Y4)

The activity of water (a_w_) performances a major role in cheese, and effects the stability, safety and quality of the ultimate product (Franks 1991). The chemical capacity of water can be obtained out by evaluating a_w_, which is connected with the enzymatic and microbial stability of the food (Grummer and Schoenfuss 2011). According to the results observed in Table 2, this could be detected that the response of the a_w_ (Y4) was significantly (P ≤ 0.05) influenced because of the basic effects of PF of protease (X1) and Protease concentration (X2) and the quadratic effect of the protease purification factor (X1^2^) and ripening time (X3^2^), also with the interaction of the protease purification factor and its concentration (X1X2), in the studied Cheddar cheeses acceleration. Additionally, it noticed that the basic and the quadratic effect of the protease PF significantly (P ≤ 0.05) influenced the a_w_ (Y4) value of Cheddar cheeses.

**Fig. 4:**
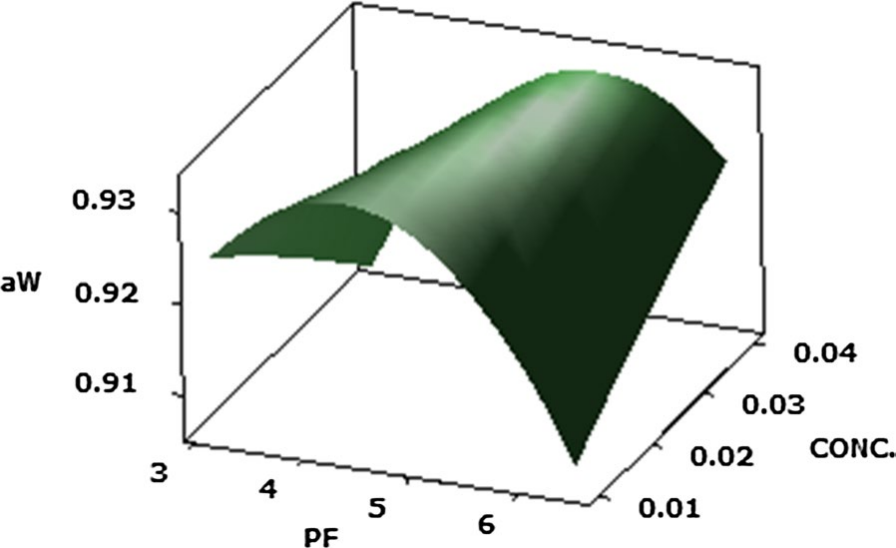
Response surface plots of the central composite design for the interaction effects of protease PF and its concentration on water activity of Cheddar cheese acceleration ripening

Figure 4 show the 3-D response surface plots for optimizing the a_w_ response of Cheddar cheeses. In this plot present that the maximal a_w_ value for the Cheddar cheese (Y4 = 0.9348).

The ideal accelerated Cheddar cheese water activity (Y4 = 0.9348) via the *P. candidum* PCA1/TT031 protease was accomplished under the following conditions—PF of protease of 3.12; protease concentration of 0.01% (v/v); and ripening time of 0.6/3 months.

#### Soluble nitrogen (SN%) (Y5)

**Fig. 5:**
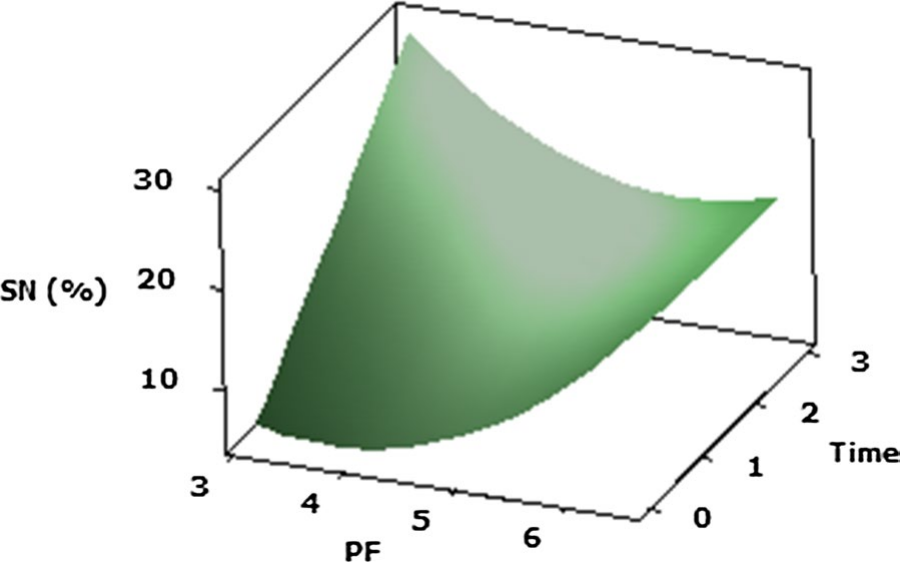
Response surface plots of the central composite design for the interaction effects of **a** protease PF and its concentration, **b** protease PF and ripening time on soluble nitrogen of Cheddar cheese acceleration ripening

The use of *P. candidum* PCA1/TT031 protease to accelerate Cheddar cheese maturation achieved in SN% values (Table 2) that were significantly (*p* ≤ 0.05) effected via the main influence of the purification factor of protease, protease concentration and ripening time of Cheddar cheese accompanied by the quadratic effect of the protease purification and protease concentration. Also, the interactions effect between the protease purification and its ripening time significantly (P ≤ 0.05) influenced the SN% of Cheddar cheeses (Y5) value. The results revealed that the most significant (P ≤ 0.05) effect on SN% (Y5) of the accelerated Cheddar cheese was produced by the quadratic impact of concentration of protease and protease purification (Table 2). Protease PF and its concentration have been confirmed to influence SN% in Cheddar cheese. For a protease PF 3.12 with a concentration of 0.01% (v/v) and ripening time 0.6/3 months, SN% was observed to be maximum (Y = 18.8%). Figure 5 shows the process by which interactions among response variables (protease PF and its concentration) significantly influence (P ≤ 0.05) the SN% of accelerated Cheddar cheese.

#### Fat (Y6)

**Fig. 6:**
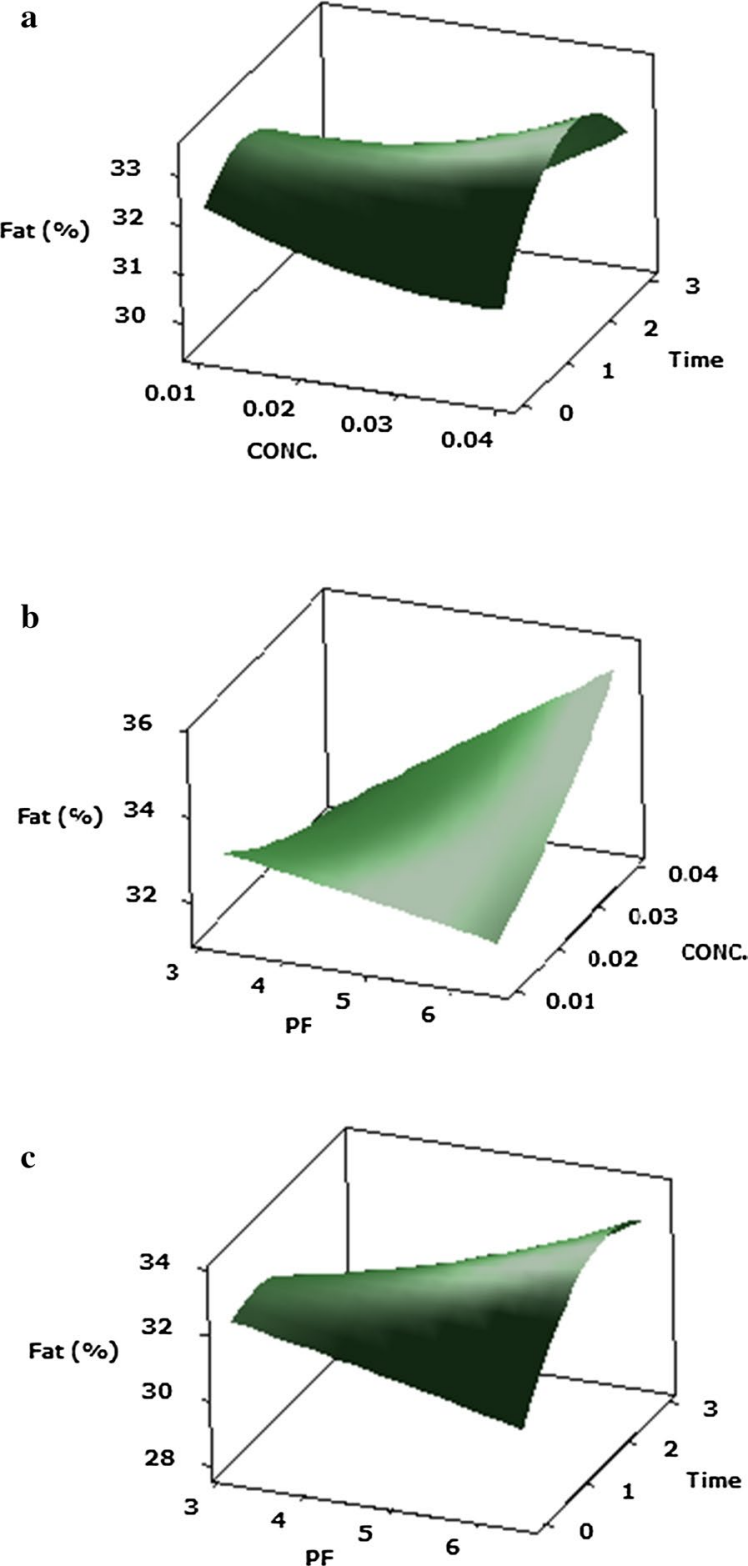
Response surface plots of the central composite design for the interaction effects of **a** protease PF and its concentration, **b** protease PF and ripening time, **c** protease concentration and ripening time on fat of Cheddar cheese acceleration ripening

Table 2 indicates that the fat (Y6) was considerably (P ≤ 0.05) influenced by the main effect of protease concentration and ripening time; the quadratic effects of ripening time; and the interactions of protease PF and protease concentration, Protease PF and ripening time and protease concentration and ripening time in the Cheddar cheeses. The outcomes displayed that the most significant (P ≤ 0.05) impact on fat value (Y6) of the Cheddar cheese was caused by the quadratic of ripening time and interactions impact of PF of protease and ripening time. It is evident from the results shown in Fig. 6a–c that the interaction effects between the protease PF-5.85 and 0.01% (v/v) protease concentration have led to a rise in the fat value (fat = 34).

#### Overall acceptability (Y7)

When employing of *P. candidum* PCA1/TT031 protease to accelerate Cheddar cheese, the primary effects of protease PF and ripening time, the quadratic results of protease concentration and ripening time, as well as the interactions of protease PF and protease concentrations, PF of protease and ripening time, all had a considerable (P ≤ 0.05) influence on response values for overall acceptability (Table 2). Main influence of ripening time, interactions between X1X2 and X1X3 were of the greatest significance (P ≤ 0.05) in overall acceptability for Cheddar cheese samples (Y7).

**Fig. 7:**
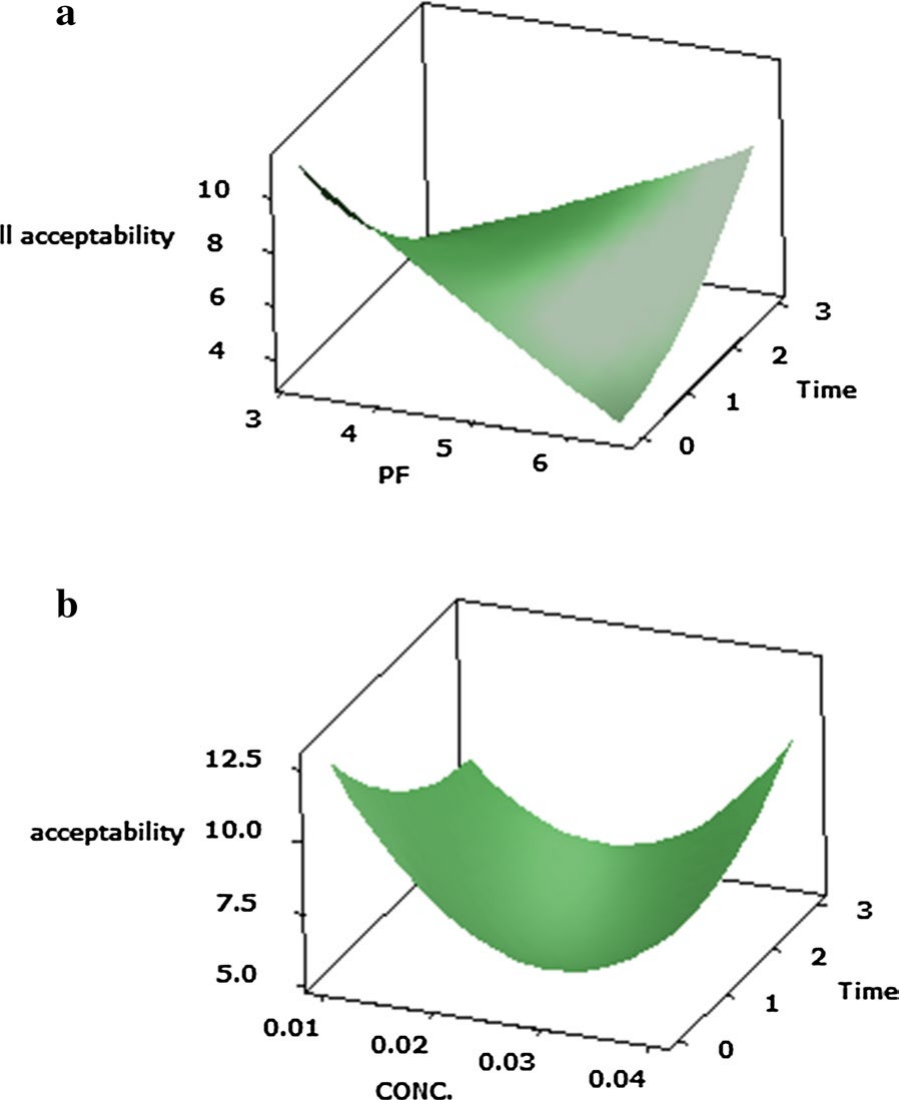
Response surface plots of the central composite design for the interaction effects of **a** protease PF and its concentration **b** protease PF and ripening time on overall acceptability of Cheddar cheese acceleration ripening

The evaluation of the overall acceptability’s curve and shape (Fig. 7a, b) shows that variations in overall acceptability (Y7) result from the non-linear functioning of overall acceptability preconditions.

#### Experimental certification of the models

The observed data and the predicted values were validated for verification of the suitability of the final response surface models. The outcomes should not show any significant difference (P > 0.05) and must be in near agreement with the observed data and the estimated values. In Additional file 1: Figure S1, it is demonstrated that the respective values of the response variables achieved from observations were closer to those estimated in the modelled equations; this proves that the RSM model is the most proper methodology for the acceleration Cheddar cheese. The acceleration plot was verified and the physiochemical characteristics were investigated. The final model was confirmed using protease PF, concentration of protease and ripening time of 3.12, 0.01% (v/v), and 0.6/3 months, respectively. It was seen that by using this model, the variables of pH, ADV, moisture, a_w,_ SN%, fat and overall acceptability was predicted as 5.4, 6.6, 35, 0.9348, 18.8, 34, and 13.6, respectively, under the optimized conditions.

### Comparison of commercial with ideal experimental Cheddar cheese

#### Free amino acids (FAA)

Generally, the occurrence of release of free amino acids in cheeses can be considered as an indicator for the incidence of the proteolytic activities. Since long, it has been observed that proteolysis is among the primary biochemical events, which occurs throughout ripening of cheese, and its derivatives such as peptides and free amino acids can reveal a main influence on the sensory properties of cheese (Calasso et al. 2015).

Exogenous peptidases and proteinases employed for accelerated ripening take into account one of the different influencing aspects essential to cheese making (Mayer and Fiechter 2013). In this study, the 17 individual free amino acids of ideal Cheddar cheese were measured. The total concentration of amino acids for the ideal cheese was 248.89 ± 0.83 mg/g and this amount is a little higher than the amount obtained with industrial cheese, which was 195.19 ± 2.59 mg/g (Additional file 2: Table S2).

The results of this study revealed that there was remarkable (P ≤ 0.05) difference in the release of 10 free amino acids in two varieties of cheese (free amino acids viz. aspartic, glutamic, aspartic, proline, threonine, valine, methionine, lysine, leucine and phenylalanine) while the other 7 free amino acids (serine, glycine, histidine, arginine, tyrosine, cysteine and isoleucine) had an insignificant (P > 0.05) impact on both cheese samples. The amount of free amino acids in ripened Cheddar cheese signifies the proteolytic activity of PF of protease and its concentration [3.2, 0.01% (v/v)] on casein decomposition.

#### Free fatty acids (FFA)

Lipolysis is the fundamental source of free fatty acids, but they are able to be created by amino acid conversion (for example, leucine and valine), or generated by the lactose metabolism (Bao et al. 2016; McCarthy et al. 2017). As with levels of individual FAA, levels of FFA found at any step of ripening are the outcome of catabolism and hydrolysis (Del Toro‐Gipson et al. 2020). The standard deviation and mean value of the corresponding percentages of individual fatty acids that existed in ideal and commercial cheeses are show in Additional file 2: Table S3.

Ethanol, 1,3-butanediol and Hexadecanoic acid exhibited the highest mean recovery (23.59 ± 0.45, 23.55 ± 0.52, 21.13 ± 0.68, respectively) in ideal cheese whereas in commercial cheese, Ethanol and 1,3-Butanediol were found to be of quantity 16.68 ± 0.34, 21.27 ± 1.42 and 15.76 ± 0.80, respectively. Hexanoic acid displayed the least average recovery in commercial and ideal Cheddar cheese (4.26 ± 0.66 and 2.37 ± 0.40, respectively).

#### Aroma profile

The cheese aroma is one of the most essential factors involving the quality and sensory characteristics of cheese. Electronic noses are not utilised to evaluate individual components after chromatographic division, but to employed the sum of volatile compounds by infusing them together into a mass spectrometer (Marilley and Casey 2004).

In this study, the qualitative estimation of the aroma profile of the cheese prepared from protease PF and its concentration (with 3.12, 0.01% in 0.6/3 months) and industrial Cheddar cheese were assessed with the electronic nose. A unique technique of this “Z” nose was to utilize two-dimensional olfactory patterns, called polar plot (Vaporprint™). This method provides visually distinguishable fragrance with a high resolution pattern (Fuchsmann et al. 2015). The VaporPrints^TM^of both kinds of cheeses are presented in Additional file 1: Figure S2a, b. As can be observed in Additional file 1: Figure S2a and b, every cheese kind had almost the same essential compounds. The characteristic nature of polar plot is dependent to the respective concentration of the numerous elements creating the mix (Marilley and Casey 2004), and so the resulting polar plot was approximately similar for every kind of cheese. There were 13 substances (a–g, reflecting different times of retention) recorded within the assessment time of 0–20 s. Among them, six were recurrent compounds for all cheese.

## Discussion

The outcomes of pH of Cheddar cheeses are in agreement with (Jung et al. 2013a, b; Hou et al. 2014), in which it was stated that increasing of pH during maturation period is attributed to the use of the formation of non-acidic decomposition products, lactic acid from lactate, and release of alkaline products decomposition and dissociated amino acids.

It can be noticed from the findings obtainable in Fig. 1a, b that the collaborations between the purification factor of protease and protease concentration has gain to a rise in the pH estimate as well as an increase in the protease purification factor and the concentration of protease. Which is a consequence of the activity of *P. candidum* PCA1/TT031 that metabolizes lactic acid and lactate (Kaminaries et al. 2019; Mane and McSweeney 2020) and causes the breakdown of curd components. Moreover, the rate of lipolysis is calculated by ADV determination. This value indicates the content of FFA dissolved in some amount of fat and it can be associated with the sensorial quality of the final products (Niro et al. 2014). Figure 2 show the 3-D response surface plots for optimizing the ADV value of Cheddar cheeses. This plot shows that the best ADV value for the Cheddar cheese (Y2 = 6.6) was observe to exist at a PF of protease 5.85, a concentration of 0.01% (v/v) and ripening time 0.6/3 months. It can also be observing that the ADV increased with an increase in the PF of protease and its concentration.

In spite of the significant (P ≤ 0.05) impact of enzyme PF on ADV value of Cheddar cheese, the main impact of ripening duration had the most significant (P ≤ 0.05) influence on the ADV content of samples. Starter enzymes throughout ripening link the ester bonds between glycerol and fatty acids in the triacylglycerides. Esterases analyse short acyl ester chains, whereas the longer acyl ester chains which are contained of more than 10 carbons were hydrolysed by lipases (Kendirci et al. 2020; Rani et al. 2019). In addition, the reduction in moisture value of Cheddar cheeses in the ripening period may be because of the biochemical variations and development of lactic acid which resulted in contraction of curd and which put an end to the aqueous stage of cheese. It also resulted in moisture loss in the ripening period (Garbowska et al. 2016). The results of moisture content of Cheddar cheese during ripening time were in an agreement with the study of (Afzaal et al. 2020; Margolies and Barbano 2018). Additionally, an increase in the protease concentration through the ripening time of Cheddar cheese caused a decrease in the Cheddar moisture content (Creamer and Olson 1982; McCarthy et al. 2016).

Despite the significant impact of the interaction of purification factor with enzyme concentration which decreased the value of a_w_, their average values remained under requirement because the water activity of cheese is generally in the range 0.70–1.00 even though most varieties have activity of water above 0.90 which is close to that of the experimental Cheddar cheese (Marcos 1993).

The reduction in a_w_ can be justified by the effect of biochemical enzyme reaction and time of ripening on cheese matrix. Compounds having higher molecular weight contain fewer molecules than compounds with lower molecular weight, resulting in a greater a_w_-reducing effect (Grummer and Schoenfuss 2011; Hickey et al. 2013). Furthermore, the results of SN (%) values conclude the effect of protease *P. candidum* PCA 1/TT031 on the casein matrix of Cheddar cheeses. Moreover, it resulted is in agreement with the study of other scientists such as Hannon et al. (2003); Jahadi and Khosravi-Darani (2017); Kaminaries et al. (2019); Nuñez et al. (1991).

Since the moisture content of samples were decrease throughout the maturation of Cheddar cheese, the fat content of Cheddar cheese was increase due to lipolysis, accompanied by a physicochemical process on Cheddar cheeses during maturation period (Soleimani-Rambod et al. 2018). The evaluation of the overall acceptability’s curve and shape (Fig. 7a, b) shows that variations in overall acceptability (Y7) result from the non-linear functioning of overall acceptability preconditions. There was a significant (*p* ≤ 0.05) increase in the overall acceptability of Cheddar cheese (Y7 = 13.6) with the addition of 0.01% (v/v) protease concentration and 3.12 PF of protease. As consequences, this enzyme of *P. candidum* PCA1/TT031 was shown to create a well-balanced equilibrium between proteolysis and lipolysis products without generating defects on flavour and texture.

In a comparison between ideal and commercial Cheddar cheese, catabolism of branched-chain, sulfur-containing FAA, and aromatic have been involved in the flavour development of cheese, resulting in the production of potent volatile aroma compounds that improve the cheese aroma (Del Toro‐Gipson et al. 2020). Exogenous proteinases and peptidases utilised for accelerated ripening take into account one of the different influencing aspects essential to cheese making (Mayer and Fiechter 2013). These outcomes are in agreement with Law and Wigmore (1983) who reported higher amount of free amino acids in the next month of ripening of Cheddar cheese using industrial neutral proteinase (neutrase) and a cocktail of no-cell extract of *Streptococcus lactis* in contrast to cheese that is not processed. In this research, the primary free amino acids in both kinds of cheese were tyrosine, glutamic acid, aspartic acid, lysine, proline, leucine, valine, phenylalanine and serine. Hannon et al. (2007) found out that the advantages of using attenuated cells of *Lactobacillus helveticus* as an extra substance in Cheddar cheese include having remarkably higher amount of amino acids, free amino nitrogen, short peptides, and development of flavour without an unfavourable effect on cheese quality or texture. The amount of FAAs can be utilised as precursors for sensory active elements (McSweeney and Sousa 2000).

The remarkable variances in the quantity of the amino acids stated earlier are probably affected not only by the proteolytic activity of concentration enzymes but also by their alteration into other products, which could influence sensory properties of cheese (Mrázek et al. 2015). High concentration of flavour components on justification of proteolysis could then produce an unlikable taste in cheese (Mrázek et al. 2015). Also, a fine balance between proteolysis and peptidolysis avoids bitterness formation in the cheese (Zhao et al. 2016). Consequently, the relative concentration and ratio of particular amino acids can significantly affect the flavour profile and texture of cheese (Eren-Vapur and Ozcan 2012; Cavanagh et al. 2014).

In addition, free fatty acids have a significant impact on aroma of cheese, either directly by their aromatic tinge, or as signs of alkanes, carbonyl compounds, alcohols and esters. Thus, free fatty acids can be a cause of cheese aroma or a cause for bad smell when they exist in high levels (Hou et al. 2014; McCarthy et al. 2017). Short chain fatty acids play a significant function directly in creating aroma in many ripened cheeses (Delgado et al. 2011; Spelbrink et al. 2015). Free fatty acids, lactones, and ethyl esters are flavouring ingredients derived from fat that effect the overall Cheddar cheese essence (Jung et al. 2013b). Bosset and Gauch (1993) investigate the volatile combinations of six kinds of cheeses and found that the flavour of these cheeses reliable not on particular key component but it was dependent on weighted concentration ratio of all compounds existed. The original influence of each volatile component to the flavour of cheeses lasts to be largely unidentified (Fernández-García et al. 2002).

Despite the significant (P ≤ 0.05) impact of some of FAA and total of FFA of commercial and ideal Cheddar cheese, the information showed that the varieties of aroma compounds released in both types of cheeses were mostly similar to each other. Aroma of cheese is counted to be the result of a balance among different volatile compounds, which individually do not exhibit the general odour (Dimitrellou et al. 2014). The results of Vapor Prints™ indicated that the stage of ripening was successfully followed by the electronic nose device, based on the volatile compounds that were found in the cheese. This outcome further verifies the suitability of the electronic nose in distinguishing the aroma compounds in cheese during its maturation period (Costa et al. 2016).

Finally, the current study exhibits the probability of manufacturing Cheddar cheese with high flavour strength in a relatively short time (0.6/3 months) by employing *P. candidum* PCA1/TT031 protease. Among the trials used in this study, favourites values were given to a PF − 3.12, 0.01% concentration of protease. This production was revealed to generate an acceptably balanced equilibrium between casein and milk fat hydrolysis products without yielding rancid, bitter or texture defect, the Cheddar cheese acquired the highest scores in all sensory characteristics.

Ideal cheese displayed a slight variation of free amino acids than industrial cheese, while free fatty acids constitution of commercial Cheddar cheese showed remarkable (P ≤ 0.05) difference in both kinds of cheese. So, the proposition of “component balance theory” among the cheese compounds is more acceptable and validated since the flavour of cheese is classified as volatile and contributes to the aroma and soluble in aqueous phase and supply to taste. Hence, the findings of the current study have a number of important implications for future manufacture of matured Cheddar cheese with similar and even higher economic advantages than normal matured cheese by decreasing the matured time to the 0.6/3 months of ripening time.

Despite, the significant (P ≤ 0.05) difference between the two cheeses in the short chain fatty acids concentration, free fatty acids and aroma profile sensory assessment did not display any difference in the general acceptability of commercial cheese and ideal cheese. Further recent biocatalysts and enhancement of suitable bio-developments, downstream routes and demonstrating researches need to be acknowledged and advanced for such an impressive objective.

## Supplementary Information

**Additional file 1: Figure S1.** Fitted line plots for predicted (Y1) and experimental values (Y0). pH (a), ADV (b), moisture content (%) (c), a_w_ (d), SN (%) (e), fat (%) (f) and overall acceptability (g) of accelerated Cheddar cheese by *P. candidum* PCA 1/TTO31 protease. **Figure S2.** Vaporprint™ of ideal (a) and commercial (b) Cheddar cheese.

**Additional file 2: Table S1.** Experimental design from the central composite design (CCD). **Table S2.** Level of individual free amino acids detected in commercial and ideal Cheddar cheese. **Table S3.** Level of individual free fatty acids detected in commercial and ideal Cheddar cheese.
