# Supplementary figures and images for: Optimizing the acceleration of Cheddar cheese ripening using response surface methodology by microbial protease without altering its quality features
Source: AMB Express. 2021 Mar 22;11:45. doi: 10.1186/s13568-021-01205-9 (PMC7984165; doi:10.1186/s13568-021-01205-9)

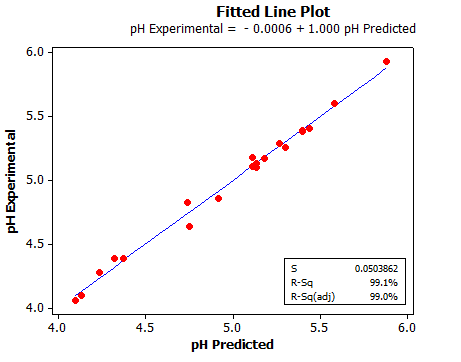

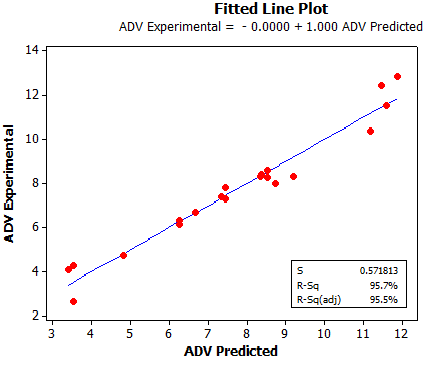

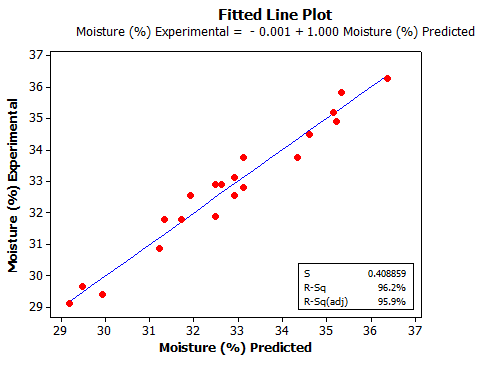

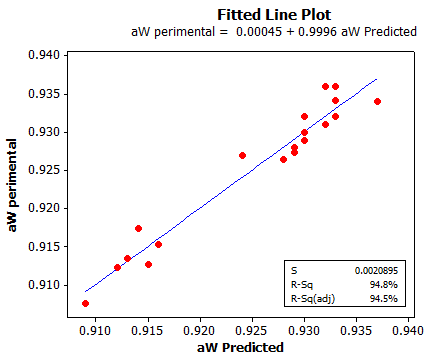

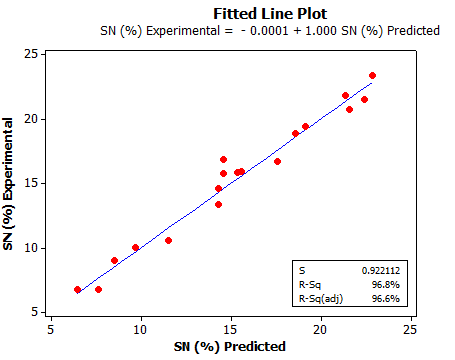

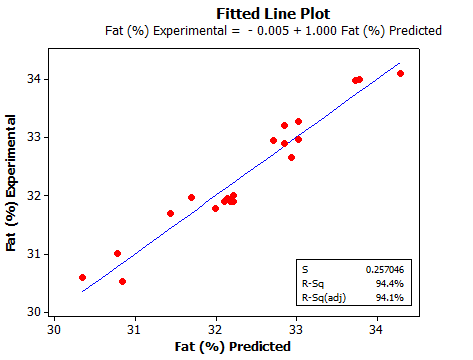

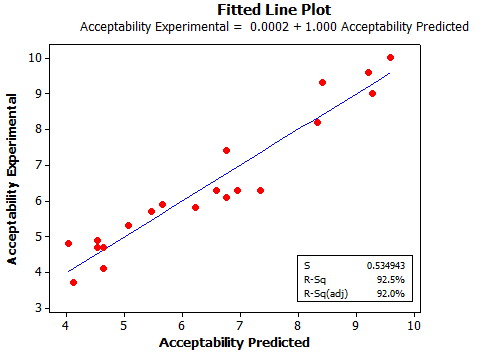


(a)

(b)

(c)

(d)

(e)

(f)

(g)

1. **Figure S1**: Amaal et al. 2020


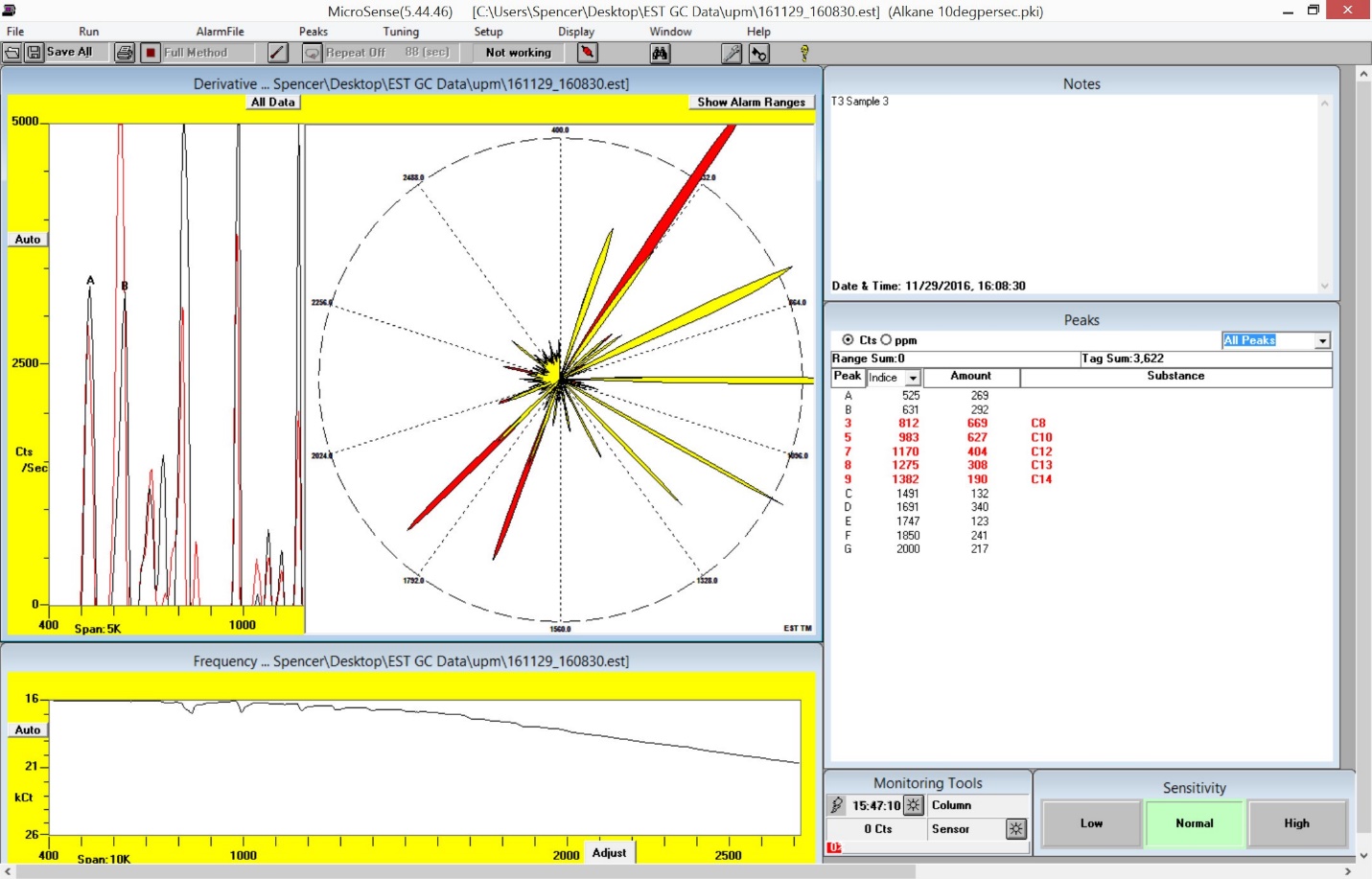

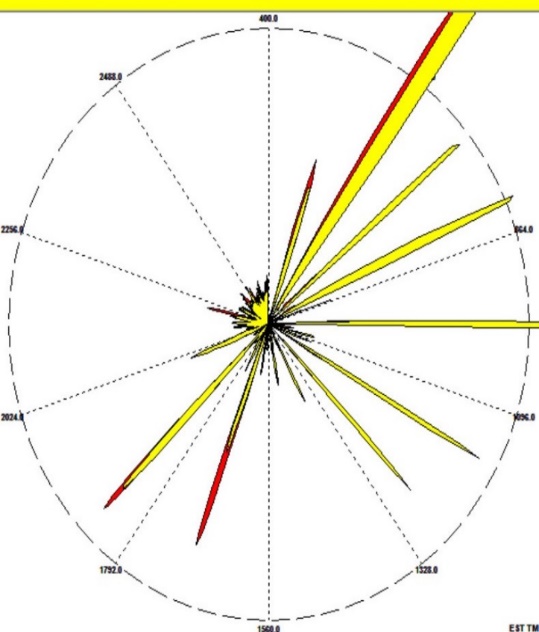


(b)

(a)

**Figure S2**:

Amaal et al. 2020

Supplement: Supplementary file 1 — Additional file 1: Figure S1. Fitted line plots for predicted (Y1) and experimental values (Y0). pH (a), ADV (b), moisture content (%) (c), aw (d), SN (%) (e), fat (%) (f) and overall acceptability (g) of accelerated Cheddar cheese by P. candidum PCA 1/TTO31 protease. Figure S2. Vaporprint™ of ideal (a) and commercial (b) Cheddar cheese. [file 13568_2021_1205_MOESM1_ESM.docx]
