# Supplementary material for: Optimizing the acceleration of Cheddar cheese ripening using response surface methodology by microbial protease without altering its quality features
Source: AMB Express. 2021 Mar 22;11:45. doi: 10.1186/s13568-021-01205-9 (PMC7984165; doi:10.1186/s13568-021-01205-9)
Supplement: Supplementary file 2 — Additional file 2: Table S1. Experimental design from the central composite design (CCD). Table S2. Level of individual free amino acids detected in commercial and ideal Cheddar cheese. Table S3. Level of individual free fatty acids detected in commercial and ideal Cheddar cheese. [file 13568_2021_1205_MOESM2_ESM.docx]

Table S1: Experimental design from the central composite design (CCD)

^c^ centre point

Amaal et al. 2020

| Run Order | Blocks | PF | Conc. protease %(v/v) | Ripening time (Month) |
| --- | --- | --- | --- | --- |
| 1 | 1 | 3.76 | 0.015 | 0.5 |
| 2 | 1 | 5.81 | 0.034 | 0.5 |
| 3 | 1 | 5.81 | 0.015 | 2.4 |
| 4 | 1 | 3.76 | 0.034 | 2.4 |
| 5^c^ | 1 | 4.79 | 0.025 | 1.5 |
| 6^c^ | 1 | 4.79 | 0.025 | 1.5 |
| 7 | 2 | 5.81 | 0.058 | 0.58 |
| 8 | 2 | 3.76 | 0.034 | 0.58 |
| 9 | 2 | 3.76 | 0.015 | 2.4 |
| 10 | 2 | 5.81 | 0.034 | 2.4 |
| 11^c^ | 2 | 4.79 | 0.025 | 1.5 |
| 12^c^ | 2 | 4.79 | 0.025 | 1.5 |
| 13 | 3 | 3.12 | 0.025 | 1.5 |
| 14 | 3 | 6.46 | 0.025 | 1.5 |
| 15 | 3 | 4.79 | 0.01 | 1.5 |
| 16 | 3 | 4.79 | 0.04 | 1.5 |
| 17 | 3 | 4.79 | 0.025 | 0.00 |
| 18 | 3 | 4.79 | 0.025 | 3 |
| 19^c^ | 3 | 4.79 | 0.025 | 1.5 |
| 20^c^ | 3 | 4.79 | 0.025 | 1.5 |

Table S2: Level of individual free amino acids detected in commercial and ideal Cheddar cheese

| Amino acid | Mean mg/g of sample | | *p-*value* |
| --- | --- | --- | --- |
|  | Ideal cheese | Commercial cheese |  |
| Aspartic acid | 17.71±0.63 | 15.92±0.13 | 0.009 |
| Glutamic | 54.45±0.94 | 45.93±0.92 | 0.000 |
| Serine | 13.14±1.01 | 12.79±1.05 | 0.701 |
| Glycine | 4.35±0.40 | 3.98±0.08 | 0.199 |
| Histidine | 6.39±0.37 | 6.07±0.06 | 0.224 |
| Arginine | 7.75±1.08 | 7.70±0.28 | 0.96 |
| Threonine | 8.82±0.23 | 7.81±0.32 | 0.01 |
| Alanine | 8.98±0.03 | 7.79±0.35 | 0.004 |
| Proline | 23.70±0.39 | 21.11±0.84 | 0.008 |
| Tyrosine | 12.95±0.33 | 1.52±0.50 | 0.06 |
| Valine | 14.47±0.43 | 12.58±0.51 | 0.008 |
| Methionine | 6.58±0.43 | 5.49±0.47 | 0.04 |
| Cysteine | 1.49±0.50 | 0.79±0.05 | 0.07 |
| Isoleucine | 11.54±0.50 | 10.52±0.48 | 0.06 |
| Leucine | 22.95±0.85 | 19.67±1.45 | 0.02 |
| Phenylanine | 13.72±0.47 | 11.80±0.23 | 0.003 |
| Lysine | 19.90±0.79 | 17.82±0.21 | 0.012 |

^*^ Note: values are reported as Mean ± standard deviation

Amaal et al. 2020

| Fatty acids | Molecular formula | Mean (Relative percentage) | | *p-*value^*^ |
| --- | --- | --- | --- | --- |
|  |  | Ideal cheese | Commercial cheese |  |
| Ethanol | C_2_H_6_O | 23.59 ± 0.45 | 16.68 ± 0.34 | 0.00 |
| 1,3-Butanediol | C_4_ H_10_O_2_ | 23.55 ± 0.52 | 21.27±1.42 | 0.05 |
| Acetic acid | C_2_H_4_O_2_ | 13.30 ± 1.13 | 10.83 ± 0.26 | 0.02 |
| 2,3- Butandiol,[S-(R*,R*)] | C_4_H_10_O_2_ | 5.63 ± 0.44 | 15.90 ± 0.51 | 0.00 |
| Botanic acid | C_4_H_8_O_2_ | 8.60 ± 0.38 | 4.93 ± 0.07 | 0.00 |
| Ammonium acetate | [C_2_H_7_NO_2_](https://pubchem.ncbi.nlm.nih.gov/#query=C2H7NO2) | 14.03±0.05 | 10.83±0.25 | 0.00 |
| Hexanoic acid-methyl ester | [C_9_H_18_O_2_](https://pubchem.ncbi.nlm.nih.gov/#query=C9H18O2) | 4.26 ± 0.66 | 2.37 ± 0.40 | 0.01 |
| Pentanoic acid | C_5_H_10_O_2_ | 6.95 ± 0.14 | 9.59 ± 0.48 | 0.00 |
| Hexadecanoic acid | C_16_H_30_O_2_ | 21.13±0.68 | 15.76±0.89 | 0.00 |

Table S3: Level of individual free fatty acids detected in commercial and ideal Cheddar cheese

^*^ Note: values are reported as Mean ± standard deviation

Amaal et al. 2020
